# Hypersensitivity reactions to high osmolality Total Parenteral Nutrition: a case report

**DOI:** 10.1186/s13223-019-0364-z

**Published:** 2019-08-30

**Authors:** Steph A. Pang, Shaun Eintracht, Jesse M. Schwartz, Belinda Lobo, Elizabeth MacNamara

**Affiliations:** 1 Faculty of Medicine, McGill University, 3655 Promenade Sir-William-Osler, Montreal, QC H3G 1Y6 Canada; 2 Division of Medical Biochemistry, Jewish General Hospital, 3755 Chemin de la Côte-Sainte-Catherine, Montreal, QC H3T 1E2 Canada; 3 Division of Allergy and Immunology, Jewish General Hospital, 3755 Chemin de la Côte-Sainte-Catherine, Montreal, QC H3T 1E2 Canada; 4 Division of Clinical Nutrition, Jewish General Hospital, 3755 Chemin de la Côte-Sainte-Catherine, Montreal, QC H3T 1E2 Canada

**Keywords:** Total Parenteral Nutrition, Osmolality, Urticaria, Hypersensitivity reactions

## Abstract

**Background:**

The full range of allergic reactions to Total Parenteral Nutrition (TPN) remains unknown. Additionally, beyond individual allergens, there may be other factors contributing to TPN hypersensitivity reactions.

**Case presentation:**

We present a case of a patient with negative skin testing to common TPN allergens who had recurrent urticarial reactions to TPN. Her skin reactions resolved once TPN was stopped. Following a literature review, we postulated that the reactions could be due to the high osmolality of her TPN. Consequently, lowering her TPN from 2785 to 1928 mOsm/kg and premedicating with cetirizine resulted in resolution of her urticaria.

**Conclusions:**

When looking at patients who have hypersensitivity reactions to TPN, one must consider that their reactions may be due to factors other than allergens. More studies are needed to clarify the relationship between high osmolality TPN infusions and non-IgE mediated hypersensitivity reactions.

## Background

While hypersensitivity to Total Parenteral Nutrition (TPN) is relatively rare, it jeopardizes critical nutritional support for patients who are otherwise unable to obtain calories and nutrients. A 2018 systematic review of 28 published cases concerning TPN hypersensitivity reactions since 1970 reveals that most manifestations are cutaneous (81.8% of cases). Frequently identified allergens come from intravenous fat emulsions, multivitamin solutions, or amino acid solutions. Currently, there is no standardized approach to determine specific allergens for patients with TPN hypersensitivity. As per published case reports, most treating teams identify causative elements by correlating the timing of introduction of components and subsequent reactions [1, 2], or by eliminating suspected offending agents and observing for symptom resolution [1, 3]. Some teams further confirm allergens by traditional skin testing [1, 4]. However, in two case reports, treating teams were unable to identify individual allergenic components [5, 6]. One report presented a patient who reacted to TPN and a lipid emulsion being given concurrently, but not when they were given separately [5]. These reports raise the question of whether interactions between TPN components may also drive hypersensitivity reactions. We now present a case of a patient with negative TPN skin testing who had recurrent urticarial reactions to TPN and lipids. We propose an etiology of TPN hypersensitivity that, to our knowledge, has not been previously considered in the literature.

## Case presentation

Our patient is a 32-year-old woman with a history of disordered eating requiring enteral feeds to supplement oral intake, in the context of gastrointestinal dysfunction of unknown cause, severe rectal prolapse requiring surgery and colostomy, and recurrent bowel obstructions. The patient was admitted in January 2018 severely cachectic requiring TPN (with Smoflipid). Initially, this was well tolerated. On Day 29, to allow for time off TPN, her TPN was increased from 90 to 110 mL/h. One hour after the TPN infusion was initiated, she developed urticaria on the neck, arms, and chest. TPN/Smoflipid were immediately held and diphenhydramine was given, with rapid improvement of pruritis and resolution of the urticaria within 3 days. She was put on lower rate TPN, which she tolerated until discharge (see Additional file 1). Skin testing for standard TPN, lipids, egg, and soy were negative. It was hypothesized that she may have reacted to niacin in the TPN, or that she had an atypical IgE hypersensitivity reaction to an unidentified allergen with late manifestations, due to daily TPN masking initial sensitization.

In May 2018, she was admitted for re-testing of TPN, with the collaboration of General Internal Medicine, Total Parental Nutrition and Allergy. She was initially started at a 1:10 dilution of standard TPN, and she did not develop reactions. The next day, her prescription was increased to undiluted standard TPN, and she developed mild urticaria on her right arm. On Day 3, lipids were started. Within 50 min, she developed erythema on her tongue, neck and face. She was given 25 mg of diphenhydramine, which improved her symptoms. The following day, she discharged herself against medical advice (see Additional file 1).

**Fig. 1:**
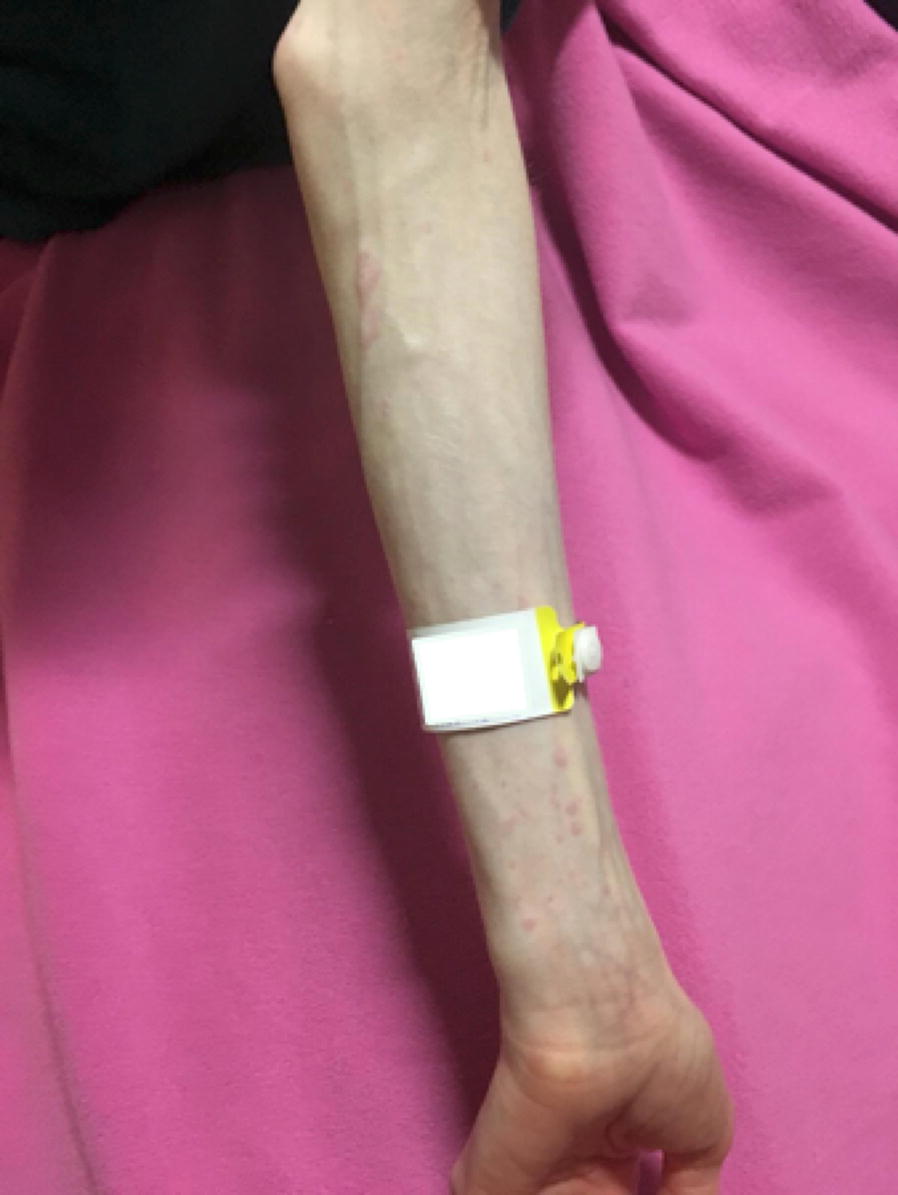
urticarial rash on left forearm. After receiving TPN at 2785 mOsmol/kg. Image courtesy of patient, used with permission. Cropping and minor lighting adjustments made for clarity
- Day 26: Her TPN rate was increased from 33 mL/h (over 24 h) to 37 mL/h (over 19 h), to provide a break off TPN.
- Day 27: While receiving TPN, she developed a pruritic rash extending to both arms, the neck, and pelvis, relieved by cetirizine.
- Day 28: It was decided to replace Smoflipid with Intralipid at 5 mL/h for 5 h, with pretreatment of 10 mg cetirizine.
- Day 29: She reported an erythematous, warm, pruritic rash on both arms. Lipids were held. Cetirizine 10 mg was given, relieving the rash.
- Day 33: A retrial 25 mL of 20% Intralipid at 5 mL/h was given with cetirizine premedication. She developed pruritis, but no urticaria.
- Day 34: Following a literature review, the allergist recommended a trial of lower osmolality TPN. TPN was re-prescribed with 400 mL of 20% amino acids, 400 mL of 70% dextrose, 400 mL of water, and standard additive doses, infused at a cyclical rate of 65 mL/h for 17 h, with 20% Intralipid infused at 2 mL/h over 5 h. This decreased the osmolality from 2785 to 1928 mOsmol/kg. With cetirizine premedication, she tolerated the diluted TPN well with no urticarial reactions.
- Day 35: Her lipids were started at 40 mL/h. She did not develop any reactions.

She was admitted again in September 2018 for severe malnutrition (Body Mass Index 11.2). Due to risk of re-feeding syndrome, initial TPN (375 kcal and 40 g a.a.) consisted of 200 mL of 20% amino acids, 100 mL of 70% dextrose, and 500 mL of water, and standard electrolytes, trace metals and vitamins, at a continuous rate of 33 mL/h. This was supplemented by oral food and Vivonex PEJ feeds. By Day 6, her TPN had been progressively advanced to 400 mL of 20% amino acids, 400 mL of 70% dextrose, standard additives, at a continuous rate of 33 mL/h. There were no reactions during this period. She tolerated her TPN well until Day 21, and the rest of her treatment proceeded as follows (see Additional file 1):

- Day 21: She was prescribed 20% Smoflipid at 5 mL/h for 6 h and cetirizine 5 mg 3 h before lipids. 2 h after starting the infusion, the TPN was temporarily infused at 100 mL/h before being lowered to the prescribed rate of 33 mL/h. 1 h afterwards, she reported itchiness, hives and erythema on both hands (see Fig. 1). Smoflipid was held. She was given one dose of diphenhydramine 25 mg. Overnight, she had abdominal pain and high volume stoma output.

She went on to receive TPN between 1391 and 1928 mOsmol/kg with cetirizine premedication for 102 days, with no further urticarial reactions.

## Discussion

We hypothesize that the origin of our patient’s dermatologic reactions was the high osmolality of the TPN. This is supported by the urticaria being provoked on 7 occasions when infusing at a high osmolality-higher rate, when compared to lower osmolality and lower rate. The diagnosis of chronic idiopathic urticaria is unlikely given the patient did not report hives when off TPN. Furthermore, her reactions are not consistent with specific IgE-mediated hypersensitivity reactions, given the negative skin tests and absence of rapid-onset multi-systemic involvement. Huston et al. reported a similar presentation of a patient who developed urticaria when TPN and lipids were administered concurrently, but not when given separately. They postulated that bisulfite in the amino acid solution may have interacted with the lipid emulsion to cause an allergic reaction [6]. However, interactions between components is an unlikely etiology for our patient’s reactions, as she still reacted to TPN without lipids.

The literature on TPN osmolality and adverse effects is limited. Small-scale studies in animals and patients have revealed that high osmolality TPN may be associated with phlebitis at venous access sites, and pulmonary and renal abnormalities [7–9]. Meanwhile, the literature on adverse skin reactions postulates that high osmolality intravenous infusions (e.g. contrast media) can cause immediate hypersensitivity reactions, independent of infusion rate [10]. Data compiled from studies between 1980 and 2009 suggest that mild to moderate hypersensitivity reactions, including urticaria and pruritis, occur in 5–13% of patients receiving high osmolality ionic contrast media; this is compared to 0.2–3% with low osmolality nonionic contrast media [11, 12]. A meta-analysis noted approximately 80% of severe non-fatal reactions with high osmolality media can be prevented by using low-osmolality media [13].

Similarly, our patient had similar symptoms (pruritis, urticaria) while on high osmolality TPN, which resolved with lower osmolality TPN and cetirizine premedication. We postulate that, similar to radiocontrast media, the osmolality of TPN may play a role in hypersensitivity reactions. Both have modifiable ionic compositions and non-physiologic osmolalities. Based on case reports and in vitro studies, mechanisms proposed in the contrast media literature include nonspecific mast cell degranulation [14], coagulation, kinin, and complement cascade activation [15], and platelet aggregation inhibition with increased serotonin release [16].

## Conclusion

We have presented the case of a 32-year-old woman who had mild generalized hypersensitivity reactions to high osmolality TPN, which did not recur when premedicated with cetirizine and switched to lower osmolality TPN. We postulate that there is a similar underlying mechanism as contrast media reactions, where the rate of immediate hypersensitivity reactions is significantly lower with low osmolality contrast media compared to high osmolality. It is important to identify preventable adverse reactions that limit TPN use for patients with life-threatening malnutrition. In addition to current methods of testing for specific allergens, the osmolality of the infusing TPN should be considered. More studies are needed to clarify the relationship between high osmolality TPN infusions and non-IgE mediated hypersensitivity reactions.

## Supplementary information

**Additional file 1.** Documented hypersensitivity reactions, associated TPN values and H1 antagonists. This table examines the patient’s hypersensitivity reactions with regards to various TPN values and H1 antagonists given.

## Data Availability

The datasets used and analysed for this case report are available from the corresponding author on reasonable request.

## Abbreviations

TPN: Total Parenteral Nutrition
PEJ: percutaneous endoscopic jejunostomy
