# Supplementary material for: Hypersensitivity reactions to high osmolality Total Parenteral Nutrition: a case report
Source: Allergy Asthma Clin Immunol. 2019 Aug 30;15:51. doi: 10.1186/s13223-019-0364-z (PMC6716805; doi:10.1186/s13223-019-0364-z)
Supplement: Supplementary file 1 — Additional file 1. Documented hypersensitivity reactions, associated TPN values and H1 antagonists. This table examines the patient’s hypersensitivity reactions with regards to various TPN values and H1 antagonists given. [file 13223_2019_364_MOESM1_ESM.pdf]

	Admission 1			Admission 2			Admission 3								
	No urticaria /pruritus	**Reaction #1**	No urticaria /pruritus	No urticaria /pruritus	**Reaction #2**	**Reaction #3**	No urticaria /pruritus	**Reaction 4**	No urticaria /pruritus	**Reaction #5**	No urticaria /pruritus	**Reaction #6**	No urticaria /pruritus	**Reaction #7**	No urticaria / pruritus
Clinical manifestations		Hives on neck, arms, and chest			Hives on right arm	Erythema on tongue, neck and face		Hives on hands Abdo pain, high volume stoma output		Hives on arms, neck, pelvis		Erythematous pruritic rash on both arms		Pruritus	
Time from infusion to reaction (h)		1			1.25	0.83		3 (infusing at incorrect elevated rate for 2h)		Unknown		30			
TPN osmolality (mOsmol/kg)	973 (initial) - 2879 (max)	2879	2879	428	1199	1199	1199 - 2785	2785	2785	2785	2785	2785	2785	2785	1391 - 1928
TPN rate (mL/h)	42 - 90	110	75	33	33	33	33	100, then 33	33-37	37	37	37	37	37	50 (min) - 122 (max)
Lipids given	Smoflipid 20% at 42mL/h x 6h	Smoflipid 20% at 42mL/h x 6h	No	No	No	(New) 20% Smoflipid at 5mL/6h	No	(New) 20% Smoflipid at 5mL/h x 6h	No	No	No	(New) 20% Intralipid at 5mL/h x 5h	20% Intralipid at 5mL/h x 5h	20% Intralipid at 5mL/h x 5h	20% Intralipid at 40mL/h, then corrected to 5mL/h x 5h
Pre-treatment H1 antagonist			Diphen-hydramine 12.mg po					Cetirizine 5mg po		Cetirizine 10mg po	Cetirizine 10mg po	Cetirizine 10mg po	Cetirizine 20mg po	Cetirizine 20mg po	Cetirizine 20mg po
Post-reaction H1 antagonist		Diphen-hydramine 12.5mg IV				Diphen-hydramine 25 mg IV		Diphen-hydramine 25 mg IV		Cetirizine 10m g		Cetirizine 10m g po			
